# Metreleptin In SHORT Syndrome With Uncontrolled Diabetes

**DOI:** 10.1210/jcemcr/luaf218

**Published:** 2025-10-09

**Authors:** Emmanouil Korakas, Aikaterini Kountouri, Ignatios Ikonomidis, Ioannis Georgiou, Baris Akinci, Vaia Lambadiari

**Affiliations:** 2nd Propaedeutic Department of Internal Medicine, Research Unit and Diabetes Center, Attikon University Hospital, School of Medicine, National and Kapodistrian University of Athens, Athens 12462, Greece; Department of Diabetes & Endocrinology, University College London Hospital NHS Foundation Trust, London NW12BU, UK; 2nd Propaedeutic Department of Internal Medicine, Research Unit and Diabetes Center, Attikon University Hospital, School of Medicine, National and Kapodistrian University of Athens, Athens 12462, Greece; 2nd Department of Cardiology, Attikon University Hospital, School of Medicine, National and Kapodistrian University of Athens, Athens 12462, Greece; Laboratory of Medical Genetics in Clinical Practice, Faculty of Medicine, School of Health Sciences, University of Ioannina, Ioannina 45110, Greece; Department of Technological Research, Izmir Biomedicine and Genome Center, Dokuz Eylül University, Izmir 35340, Türkiye; 2nd Propaedeutic Department of Internal Medicine, Research Unit and Diabetes Center, Attikon University Hospital, School of Medicine, National and Kapodistrian University of Athens, Athens 12462, Greece

**Keywords:** metreleptin, insulin resistance, lipodystrophy, SHORT syndrome

## Abstract

We report a case of metreleptin use in a patient with SHORT syndrome, a rare congenital disorder characterized by partial lipodystrophy, distinctive dysmorphic features, and severe insulin resistance. The patient was initially misdiagnosed with type 1 diabetes in early childhood because of insulin dependence, but genetic testing later confirmed a pathogenic *PIK3R1* variant consistent with SHORT syndrome. Despite high-dose insulin therapy and adjunctive agents, glycemic control remained poor. Introduction of metreleptin led to a rapid and sustained improvement in glycemic control, with a marked reduction in insulin requirements. Notably, the patient did not exhibit hepatic steatosis or hypertriglyceridemia—features typically associated with metreleptin-responsive lipodystrophy—suggesting a broader therapeutic potential of the drug. Treatment interruption due to weight issues resulted in swift metabolic deterioration, including a sharp rise in glycosylated hemoglobin and increased insulin needs. Upon reinitiation, metabolic parameters rapidly improved. This case highlights the potential utility of metreleptin in treating severe insulin resistance in SHORT syndrome, even in the absence of classical metabolic complications. Further studies are needed to define its role in atypical lipodystrophy syndromes with unusual metabolic phenotypes.

## Introduction

SHORT syndrome (OMIM #269880) is a rare congenital disorder defined by multiple anomalies including short stature, hyperextensibility of joints, ocular depression, Rieger anomaly, and teething delay, from which the acronym derives [1]. Other features include intrauterine growth retardation, a progeroid facial appearance, sensorineural hearing loss, partial or generalized lipodystrophy, and severe insulin resistance [2]. Fewer than 50 cases had been reported in the literature by 2016, and specific diagnostic criteria remain undefined. Most cases are linked to mutations in the *PIK3R1* gene, disrupting phosphatidylinositol 3-kinase signaling—a key pathway in insulin action and adipocyte differentiation [3, 4]. Insulin resistance in SHORT syndrome typically emerges during adolescence and can lead to early-onset diabetes mellitus, hypertriglyceridemia, and nonalcoholic fatty liver disease. Standard interventions often prove inadequate because of the underlying molecular defect. Lipodystrophy, frequently present in these patients, further impairs metabolic homeostasis by promoting ectopic fat deposition and lipotoxicity.

Lipodystrophies are rare disorders marked by partial or complete loss of adipose tissue [5]. They are categorized by distribution (generalized vs partial) and etiology (genetic vs acquired) [6]. A common hallmark is severe insulin resistance, often accompanied by hypertriglyceridemia, fatty liver, and premature cardiovascular disease. Metreleptin, a recombinant leptin analogue, is the only approved therapy for generalized and partial forms [7]. It improves insulin sensitivity, reduces insulin requirements, and lowers triglyceride levels [8]. The mechanisms are various and have been investigated thoroughly both in preclinical and clinical settings. In the hypothalamus, metreleptin restores leptin signaling, reducing hyperphagia and energy intake. Peripherally, metreleptin promotes fatty acid oxidation and decreases ectopic fat deposition in liver and muscle, improving insulin signaling. It enhances insulin-mediated suppression of hepatic gluconeogenesis and increases glucose uptake in skeletal muscle. Although metreleptin is effective in lipodystrophic syndromes, its role in SHORT syndrome has not been previously studied. Here, we present a case of a patient with SHORT syndrome treated with metreleptin for refractory insulin resistance.

## Case Presentation

This 22-year-old man was delivered by caesarean section at 35 weeks' gestation with a birth weight of 2100 g. His parents were nonconsanguineous and neither displayed signs or features consistent with SHORT syndrome. Aside from hypospadias noted in infancy, his medical history was unremarkable until age 15 years, when elevated fasting glucose (124 mg/dL) (SI: 6.9 mmol/L) (normal reference range, 70-99 mg/dL [SI: 3.9-5.5 mmol/L]) was detected during routine screening. An oral glucose tolerance test confirmed diabetes mellitus with marked postprandial insulin resistance (Table 1).

**Table 1.** Oral glucose tolerance test at the age of 15 years old

	0 minutes	30 minutes	60 minutes	90 minutes	120 minutes	150 minutes	180 minutes
**Glucose**	104 mg/dL (5.8 mmol/L)	279 mg/dL (15.5 mmol/L)	400 mg/dL (22.2 mmol/L)	432 mg/dL (24.0 mmol/L)	430 mg/dL (23.9 mmol/L)	388 mg/dL (21.5 mmol/L)	315 mg/dL (17.5 mmol/L)
**Insulin**	7.75 μIU/mL (53.8 pmol/L)	49.08 μIU/mL (340.8 pmol/L)	130.4 μIU/mL (905.3 pmol/L)	167 μIU/mL (1158.7 pmol/L)	139.1 μIU/mL (966.8 pmol/L)	212.6 μIU/mL (1475.5 pmol/L)	131.7 μIU/mL (914.4 pmol/L)

Glutamic acid decarboxylase 65-kilodalton isoform and islet antigen 2 antibodies were negative. Initial glycosylated hemoglobin (HbA1c) was 8.2% (66 mmol/mol) (normal reference range, 4.0-5.6% [SI: 20-42 mmol/mol]). Hormonal and lipid profile was unremarkable. At presentation, the patient did not report classical catabolic symptoms such as polyuria, polydipsia, polyphagia, or weight loss.

The patient had a progeroid appearance, the height was 165 cm (25th percentile), weight was 39.5 kg (<3rd percentile), pubic hair was Tanner Stage IV, axillary hair was Tanner Stage II-III, and testes volume was 15 mL bilaterally. Mandibular hypoplasia (micrognathia) and pectus carinatum were also noted.

The patient was misdiagnosed as having type 1 diabetes. He was started on glargine and lispro insulin and seemed to achieve a fair glycemic control for some years (HbA1c 6.7%-7.2%) (normal reference range, 4.0%-5.6% [SI: 20-42 mmol/mol]). His weight, however, remained below normal range despite adequate food consumption, without findings of malabsorption. At the age of 18 years, the patient was on glargine U-300 22 units nightly, and a total lispro unit count of up to 40 units daily, with low body mass index (15 kg/m^2^).

## Diagnostic Assessment

During his physical examination, a profound loss of adipose tissue, especially in the upper and lower limbs, was detected, along with substantial muscular atrophy and weakness. Insulin-to-carbohydrate ratio was 1:7.5 at the current stage, and overall glycemic control was poor (HbA1c: 8.7%, (72 mmol/mol) (normal reference range, 4.0%-5.6% [SI: 20-42 mmol/mol]). Low serum leptin levels were detected (4 ng/mL; 0.25 nmol/L; reference range for males, 0.5-12.5 ng/mL [0.03-0.78 nmol/L]).

Whole-exome sequencing identified a pathogenic *PIK3R1* variant (NM_181523.3:c.1945C > T:p.(Arg649Trp)), confirming the diagnosis of SHORT syndrome.

## Treatment

A lipodystrophy diagnosis was established and metreleptin 5 mg once daily was initiated.

## Outcome and Follow-up

The patient showed rapid improvement in glycemic control. After 3 months, his glargine dose decreased to 12 units daily, with only occasional fast-acting insulin use. At 9 months, HbA1c was 6.7% (50 mmol/mol) (normal reference range, 4.0%-5.6% [SI: 20-42 mmol/mol]), and basal insulin was further reduced to 6 units. However, because of reduced appetite and gradual weight loss, the patient chose to stop metreleptin. This led to significant metabolic deterioration over 6 months, with fasting blood glucose of 325 mg/dL (7.8 mmol/L, normal reference range 70-99 mg/dL [SI: 3.9-5.5 mmol/mol], HbA1c of 9.3% (78 mmol/mol) (normal reference range, 4.0%-5.6% [SI: 20-42 mmol/mol]), glargine U-300 at 18 units daily, and increased lispro requirements. Metreleptin was reinitiated alongside dietary advice and protein supplementation. The course of the follow-up is summarized in Table 2.

**Table 2.** Anthropometric and metabolic traits of the patient during the course of his 36 months of follow-up

	Baseline	3 months	9 months	18 months	24 months (6 months off treatment)	33 months (1 month after reinitiation)	36 months	Reference ranges
**FBG**	141 mg/dL (7.8 mmol/L)	—	97 mg/dL (5.4 mmol/L)	—	325 mg/dL (18.0 mmol/L)	236 mg/dL (13.1 mmol/L)	138 mg/dL (7.7 mmol/L)	70-99 mg/dL (3.9-5.5 mmol/L)
**HbA1c**	8.7% (72 mmol/mol)	—	6.7% (50 mmol/mol)	6.3% (45 mmol/mol)	9.3% (78 mmol/mol)	8.1% (65 mmol/mol)	7% (53 mmol/mol)	4%-5.6% (20-42 mol/mol)
**Body weight**	42.9 kg	—	39 kg	37.9 kg	42 kg	41 kg	41 kg	N/A
**Glargine U-300 daily dose**	22 units	12 units	6 units	8 units	18 units	20 units	10 units	N/A
**TC**	143 mg/dL (3.70 mmol/L)	—	154 mg/dL (3.99 mmol/L)	—	—	—	175 mg/dL (4.53 mmol/L)	<200 mg/dL < 5.17 mmol/L
**LDL-C**	76 mg/dL (1.97 mmol/L)	—	91 mg/dL (2.36 mmol/L)	—	—	—	104 mg/dL (2.69 mmol/L)	< 100 mg/dL < 2.58 mmol/
**HDL-C mg/dL** **(mmol/L)**	36 mg/dL (0.93)	—	45 mg/dL (1.16)	—	—	—	41 mg/dL (1.06)	>60 mg/dL > 1.55 mmol/L
**TGs**	157 mg/dL (1.78 mmol/L)	—	91 mg/dL (1.03 mmol/L)	—	—	—	151 mg/dL (1.71 mmol/L)	<150 mg/dL < 1.7 mmol/L

Abbreviations: FBG, fasting blood glucose; HbA1c, glycosylated hemoglobin; HDL-C: high-density lipoprotein cholesterol; LDL-C, low-density lipoprotein cholesterol; TC, total cholesterol; TGs: triglycerides.

## Discussion

This is a case report of metreleptin treatment in a patient with SHORT syndrome, lipodystrophy, and severe insulin resistance, misdiagnosed as type 1 diabetes. Metreleptin significantly ameliorated glycemic control, with the benefits being sustainable as long as the patient received metreleptin treatment, and immediately reemerging as soon as treatment was reinitiated.

SHORT syndrome remains a rare condition, with no more than 50 cases having been reported in the literature until recently [2]. SHORT syndrome is caused by mutations in the *PIK3R1* gene (5q13.1), which encodes phosphatidylinositol 3-kinase regulatory subunit p85α that, in turn, plays an important role in the growth and maturation of various cell types like adipocytes. The first report that directly associated SHORT syndrome with mutations in the *PIK3R1* gene was by Chudasama et al [4] in 2013. Although the syndrome's acronym reflects common traits—short stature, joint hyperextensibility, ocular depression, Rieger anomaly, and teething delay—the phenotype is highly variable. However, features like micrognathia, low-set ears, triangular face, and lipodystrophy are present in >90% of cases [9].

Beyond physical traits, the patient's main issue was severe postprandial insulin resistance, a common feature in SHORT syndrome [3, 4]. Masunaga et al [10] reported diabetes onset at puberty in a girl with an HbA1c of 9.2%, despite stopping GH, which is known to impair glucose tolerance. However, literature suggests GH use is not linked to diabetes development in SHORT syndrome. As in our case, insulin resistance typically occurs without dyslipidemia. Glycemic control on insulin is generally poor, regardless of age at onset. An exception was reported by Yin et al [11], where metformin and pioglitazone improved insulin resistance in an 11-year-old boy who had not yet developed diabetes. Zhang et al [9] described mixed responses to standard treatments: 2 patients initially failed insulin therapy via continuous subcutaneous insulin infusion or lispro but later maintained euglycemia on metformin 750 mg daily after stopping insulin. Following a similar report by Kawana et al [12], Hamaguchi et al [13] reported a case where addition of dapagliflozin alleviated insulin resistance and reduced daily insulin requirement in a young female patient with SHORT syndrome. However, fat tissue was not severely decreased in this patient, contrary to ours, and body weight was decreased by 2 to 3 kg during dapagliflozin treatment, an effect completely undesirable by our patient. Because of concerns about weight loss, potential diabetic ketoacidosis during insulin dose reduction, and adverse gastrointestinal effects, metformin or sodium–glucose cotransporter 2 inhibitors were not considered in our patient.

Severe insulin resistance in SHORT syndrome stems from the underlying *PIK3R1* mutation. *PIK3R1* plays a key role in insulin receptor (*INSR*) signalling, and pathogenic variants like p.Arg649Trp impair phosphatidylinositol 3-kinase-AKT pathway activation [14]. Monogenic defects in proximal insulin signalling—such as *INSR* mutations—cause severe hyperinsulinemia and hyperglycemia but lack features like hypertriglyceridemia and hepatic steatosis [15]. In contrast, AKT serine/threonine kinase 2 (*AKT2)* mutations result in significant dyslipidemia and high liver fat. The insulin resistance phenotype in SHORT syndrome, involving a defect downstream of *INSR* but upstream of *AKT2*, closely resembles that seen in *INSR*-related syndromes.

Metreleptin is an analogue of human leptin that binds to and activates leptin receptor, thereby imitating the physiological effects of the endogenous hormone. It is the only officially indicated treatment for generalized and partial lipodystrophy, and leads to sustained decreases in HbA1c and, subsequently, in daily insulin requirements [16-18]. Metreleptin treatment for insulin resistance has not been studied specifically in patients with SHORT syndrome. Most relevant data regarding the effect of metreleptin in rare causes of insulin receptor-mediated insulin resistance come from patients with Rabson-Mendenhall syndrome (RMS) [19, 20]. RMS is caused by homozygous or compound heterozygous *INSR* mutations. Recombinant methionyl human leptin (r-metHuLeptin) was first used nearly 20 years ago in 2 siblings with RMS, leading to 40% to 60% reductions in fasting glucose and insulin and improved HbA1c after 10 months [21]. Okawa et al [20] reported a 1.4% HbA1c drop with metreleptin in RMS, though insulin needs rose over time, similar to findings by Brown et al [19]. As in our case, glycemic control worsened upon treatment cessation. This may relate to metreleptin's effect on energy intake, a mechanism possibly shared in SHORT syndrome. Other actions, like glucagon suppression or β-cell effects, seem less likely in RMS and receptoropathies.

In conclusion, metreleptin treatment in SHORT syndrome resulted in sustained glycemic improvement despite unclear mechanisms. Given poor responses to standard therapies, this case supports metreleptin's potential role in managing severe insulin resistance in SHORT syndrome and related lipodystrophies. Further studies are needed to validate its efficacy and better define its place in rare metabolic disorders.

## Learning Points

- SHORT syndrome should be considered in young patients with severe insulin resistance and lipodystrophic features, even if previously misdiagnosed with type 1 diabetes.
- Metreleptin can significantly improve glycemic control and reduce insulin requirements in patients with SHORT syndrome, even in the absence of hypertriglyceridemia or hepatic steatosis.
- The insulin resistance phenotype in SHORT syndrome appears mechanistically similar to insulin receptoropathies, supporting the rationale for leptin-based therapies in selected genetic forms of insulin resistance.

## Contributors

All authors made individual contributions to authorship. V.L, E.K., A.K. and I.I. were involved in the diagnosis and management of the patient. E.K. was responsible for drafting and submission of the manuscript. V.L. and B.A. revised the manuscript for important intellectual content. I.G. was responsible for the genetic testing of the patient. All authors reviewed and approved the final draft.

## Data Availability

Original data generated and analyzed for this case report are included in this published article.
